# Bone Marrow Transplantation in Patients With Acute Leukemia In Cuba: Results From the Last 30 Years and New Opportunities Through International Collaboration

**DOI:** 10.1200/JGO.18.00109

**Published:** 2018-11-30

**Authors:** José Carnot Uria, Calixto Hernández Cruz, Jorge Muñío Perurena, Wilfredo Torres Yribar, Jesús Diego de la Campa, Concepción del Castillo Carrillo, Yusaima Rodríguez Fraga, Julio A. López Silva, Kali Cepero Llauger, Ibis K. Pardo Ramírez, Aliette García García, Karen Sweiss, Pritesh R. Patel, Damiano Rondelli

**Affiliations:** **José Carnot Uria**, **Calixto Hernández Cruz**, **Jorge Muñío Perurena**, **Wilfredo Torres Yribar**, **Jesús Diego de la Campa**, **Concepción del Castillo Carrillo**, **Yusaima Rodríguez Fraga**, **Julio A. López Silva**, **Kali Cepero Llauger**, **Ibis K. Pardo Ramírez**, **Aliette García García**, Universidad Hermanos Ameijeiras Hospital, Havana, Cuba; and **Karen Sweiss**, **Pritesh R. Patel**, and **Damiano Rondelli**, University of Illinois at Chicago, Chicago, IL.

## Abstract

Blood and marrow transplantation (BMT) has been performed in Cuba for over 30 years with limited resources and without international relationships. Researchers from University of Illinois at Chicago and Hermanos Ameijeiras Hospital (HAH) in Havana collaborated on retrospectively analyzing 101 consecutive patients with adult acute leukemia who received BMT at HAH from June 1986 to January 2016. Of these, 82 had acute myeloid leukemia (AML) and 19 had acute lymphoblastic leukemia (ALL). BMT eligibility criteria included prior morphologic complete remission, no severe comorbidities, and age between 16 and 60 years. Patients with an HLA-matched donor received an allogeneic BMT, whereas the others received an autologous BMT. All patients received fresh stem cells from marrow (80%) or mobilized peripheral blood (19%). Of 82 patients with AML, 35 received an allogeneic (AML-allo) and 47 an autologous (AML-auto) BMT. Both groups had comparable median age (37 years) and follow-up of survivors. Overall survival (OS) was 34% in AML-allo and 38% in AML-auto. The transplant-related mortality rate was 40% in AML-allo and 17% in AML-auto, whereas the relapse-related mortality rates were 25% and 40%, respectively. Of the 19 patients with ALL, six received an allogeneic transplant. Of these, transplant-related mortality occurred in one patient and three died after disease relapse (OS, 33%). Of 13 patients who received autologous transplants, transplant-related mortality occurred in three and six died after disease relapse (OS, 31%). To our knowledge, this is the first scientific report on BMT performed in patients with acute leukemia in Cuba. The collaboration between University of Illinois at Chicago and HAH will further develop capacity building in research and implementation of new diagnostic and therapeutic strategies in Cuba.

## INTRODUCTION

In low- and middle-income countries (LMICs), patients often have limited access to blood cancer care and still less access to bone marrow transplantation (BMT), because of lack of infrastructures, health care providers, or unaffordable costs.^1^ In contrast with many other LMICs, in Cuba, a country with approximately 11 million inhabitants and a low income per capita, the government provides free health care to every citizen,^2^ including cancer treatment and BMT. This procedure is available in five centers on the island: three in Havana and the others in Villa Clara and Holguín. However, allogeneic BMT (allo-BMT) is only available in Havana.

Although Cuba is planning to increase its BMT program, the total number of procedures is still low, due to multiple limiting factors. One of these is the difficulty in acquiring medicines, materials, and equipment, which has been affected by the economic embargo imposed on Cuba. Therapeutic agents commonly used in reduced-intensity conditioning regimens that would allow BMT in patients older than 60 years have not been available in Cuba. Peripheral blood hematopoietic cells can be only collected and used in the centers of the capital, because apheresis machines are not available outside Havana. Because stem cell cryopreservation systems are not available in the country, all transplantations must be carried out using fresh hematopoietic progenitor cells. Therefore, in autologous transplants, the stem cells are collected from the patient and kept at 4°C for 48 to 72 hours, thus preventing the possibility of using conditioning regimens lasting longer than 3 days.^3^ Another limiting factor has been the inability of Cuban centers to access international marrow-donor registries for patients lacking a matched sibling and, until a few years ago, to establish scientific exchanges with centers in the United States and Europe.

The Hermanos Ameijeiras Hospital (HAH) in Havana is the largest tertiary health care institution for adult patients in Cuba. The Hematology Service includes eight hematologists who attend 27 inpatient beds. Of these beds, four are dedicated only to acute leukemia and three to ongoing BMT. Despite the cited difficulties and the limited resources, the HAH BMT program started in 1986 and has been performing, on average, 15 to 18 autologous or matched related-donor allogeneic BMTs yearly (C. Hernández Cruz, personal communication, 2015).

In 2016, the BMT Program at the University of Illinois at Chicago (UIC) initiated collaborating with the BMT Program at HAH. Main objectives of this collaboration have been (1) training a Cuban physician in novel BMT strategies such as reduced-intensity allogeneic stem cell transplantation, autologous transplant in elderly patients with myeloma, and use of haploidentical donors with nonmyeloablative regimens; (2) organizing a training conference in Havana for Cuban physicians attending BMT units throughout the country; and (3) building research capacity and supporting analysis of clinical outcome in BMT in Cuba.

The first objective was achieved in 2016 by hosting a member of the HAH BMT staff for 3 months as an observer of all the components of the UIC BMT Program. Upon his return, HAH officially implemented a haploidentical transplant program and started performing BMT in patients older than 60 years. The second objective was achieved by co-organizing the BMT conference in Havana in October 2017, where updated protocols and results of BMT were presented by members of UIC and HAH to more than 70 participants who came from different parts of the island. The third objective was initially addressed by collaborating on the design and analysis of the retrospective study presented here.

## PATIENTS AND METHODS

A retrospective study by chart review was approved by the HAH Institutional Review Board to analyze the results of BMT from June 1986 to January 2016 in patients with adult acute leukemia who underwent BMT . Data collection was limited by the few BMT details in the records stored from decades ago and by scarcity of clinical information on follow-up encounters for many patients. However, the Cuban BMT team was able to obtain information on diagnosis, patients’ sex and age, type of transplant, source of stem cells, overall survival (OS), and rates of transplant-related mortality (TRM) versus relapse-related mortality.

A total of 101 consecutive cases were analyzed. Eligibility criteria for autologous and allogeneic BMT included age older than 16 years and younger than 60 years, achievement of morphologic complete remission, and no severe organ impairment before BMT. Patients who had an HLA-matched related donor received an allo-BMT, whereas all the others received an autologous BMT (auto-BMT). Cytogenetic and molecular analyses at diagnosis were not performed in most patients because those analyses were not yet available for patients receiving their diagnosis in the 1980s and because these studies are still not available routinely in Cuba.

### Transplants

In allo-BMT, matched related-donor selection was based on HLA class I and II matching. From 1985 to 2010, class I HLA A and B antigens were typed by serologic assays, and class II DR antigen was tested by mixed leukocyte reaction and serologic assays. In 2011, low-resolution molecular typing was introduced (ie, HLA, A, B, C, DRB1, and DQB1), and since 2014, high-resolution molecular HLA typing is available. In both auto- and allo-BMT, the sources of hematopoietic stem cells were bone marrow or peripheral blood stem cells. These were infused fresh within 72 hours after collection. Conditioning regimens used in auto- and allo-BMT were standard myeloablative oral busulfan and intravenous cyclophosphamide (Cy), total-body irradiation (TBI) and Cy or TBI, Cy, and VP16.^4,5^ In these regimens, Cy was administered in 1 day, and in patients undergoing autologous BMT, this would be infused on the same day of marrow harvest. TBI, 10 Gy, was initially administered as single fraction and, for most patients, in two daily 5-Gy fractions. In allo-BMT, graft-versus-host disease (GVHD) prophylaxis was standard cyclosporine-A and methotrexate administered on days 1, 3, 6, and 11 after transplant.

### Statistical analysis

Cumulative incidence and survival were analyzed using Kaplan-Meier statistics. Differences within AML and ALL groups were analyzed by *t* test or χ^2^ test. Statistical tests were performed using GraphPad Prism, version 7.0 (GraphPad Software, San Diego, CA).

## RESULTS

### Transplant policy for auto- and allo-BMT in patients with AML

Patients’ characteristics related to age, diagnosis, stem cell source, and time of follow-up are listed in Table 1. Because of the lack of cryopreservation facilities in Cuba, 90% of the transplantations were performed using fresh marrow cells, and 10% were performed using peripheral blood stem cells. In auto-BMT, the conditioning regimens were administered within 72 hours from stem cell collection. Of 101 patients, 83 had AML. Of these, 35 received an allogeneic (AML-allo) and 47 an autologous (AML-auto) BMT. No differences were recorded between patients’ characteristics receiving allo- or auto-BMT. The median age of the patients was 37 (range, 22 to 54) years in the AML-allo group and 36 (range, 18 to 58) years in the AML-auto group. Of 19 patients with ALL, six received an allogeneic (ALL-allo) and 13 received an autologous (ALL-auto) BMT. The median age in the two groups was 36 years (range, 18 to 47 years) and 27 years (range, 17 to 34 years), respectively. Because of the policy established at HAH, all eligible patients with ALL underwent either auto- or allo-BMT only on the basis of matched related donor availability.

**Table 1:**
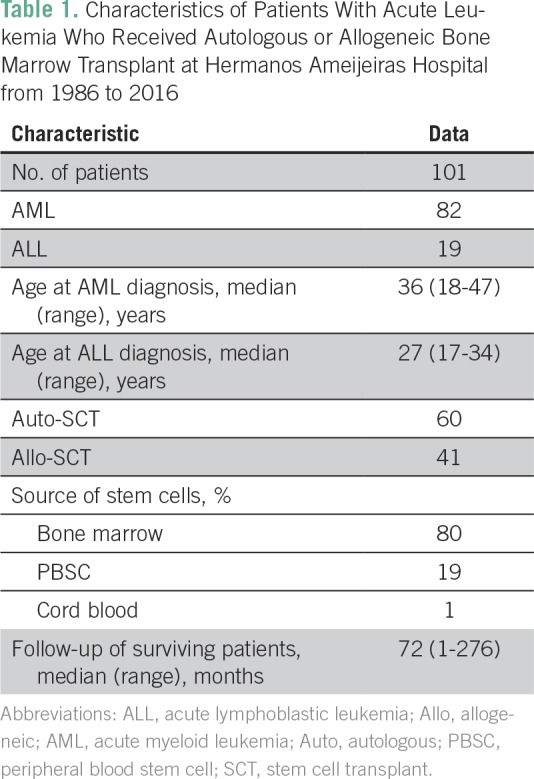
Characteristics of Patients With Acute Leukemia Who Received Autologous or Allogeneic Bone Marrow Transplant at Hermanos Ameijeiras Hospital from 1986 to 2016

### Comparable Survival in auto- and allo-BMT

The median follow-up for surviving patients in the study is 72 months (range, 1 to 206 months). Median survival for patients with AML is 69 months (range, 1 to 206 months) and 92 months (range, 15 to 276 months) for patients with ALL (*P* = .4). The limited follow-up is in part due to the policy that patients from outside Havana return to their primary hematologist within 1 to 2 years after transplantation, or, in some cases, because patients left Cuba soon after transplantation for personal or logistic reasons. Incidence of acute and chronic GVHD was not analyzed because these data were not available in the records. However, deaths related to GVHD, as well as infections or organ dysfunction after transplantation had been all identified as causes of TRM.

**Fig 1:**
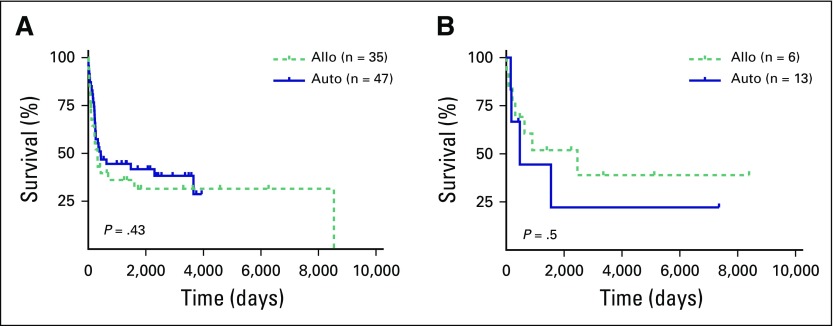
Survival in 82 patients with AML and 19 patients with ALL after bone marrow transplantation in Cuba. Cumulative overall survival in (A) patients with AML who received an allogeneic (n = 35) or autologous (n = 47) transplant and (B) patients with ALL who received an allogeneic (n = 6) or autologous (n = 13) transplant. Differences in survival were not statistically significant. ALL, acute lymphoblastic leukemia; allo, allogeneic; AML, acute myeloid leukemia; auto, autologous.

Of the 82 patients with AML who received BMT, 38% of those receiving an auto-BMT and 34% of those receiving an allo-BMT survived. However, auto-BMT was associated with higher relapse-related mortality but a lower TRM rate, compared with those receiving allo-BMT (Table 2). Of 13 ALL-auto cases, nine patients died: Six died after disease relapse and TRM occurred in three (OS, 31%), whereas of six patients who underwent ALL-allo, three died after disease relapse and TRM occurred in one (OS, 33%; Table 2). OS curves for patients with AML and those with ALL, as well for recipients of allogeneic and autologous hematopoietic BMT in each group are shown in Figure 1. These results show comparable outcome in auto- and allo-BMT in adult patients with acute leukemia who underwent transplantation in Havana.

**Table 2:**
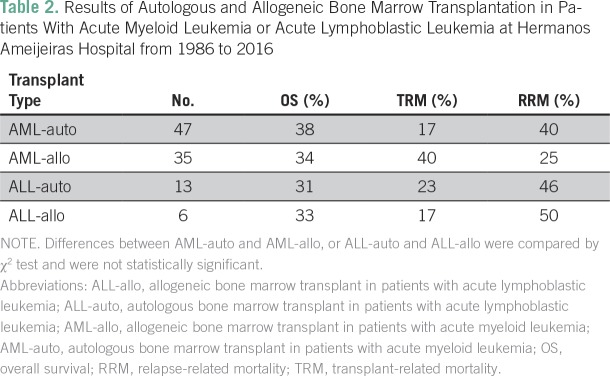
Results of Autologous and Allogeneic Bone Marrow Transplantation in Patients With Acute Myeloid Leukemia or Acute Lymphoblastic Leukemia at Hermanos Ameijeiras Hospital from 1986 to 2016

## DISCUSSION

To our knowledge, this is the first Cuban experience of BMT reported outside of Cuba and is a useful source of information to many investigators to better understand the policies and outcomes of patients with leukemia in a country with limited resources and international connections. The collaboration between UIC and HAH in Havana has created a new opportunity for building capacity in research, clinical strategy development, and education. A recent report on emerging BMT centers in Central or South America did not include any data from Cuba^6^; however, it showed that in other Latin American countries, there is a low rate of BMT compared with North America. Socioeconomic factors such as low per capita gross national income, low per capita health care expenditure, and low BMT team density were reported as possible explanations of their findings. We envision that despite the limited financial resources, other factors will likely contribute to the expansion of BMT in LMIC. In fact, our experience, like those of others in southeast Asia, suggests the following: (1) an international network allows LMIC teams to learn and build comprehensive transplant programs^7^; (2) use of generic drugs, fast cryopreservation procedures,^8,9^ or outpatient-based BMT can lower the cost of BMT^10,11^; (3) increasing knowledge of reduced-intensity BMT regimens will allow LMIC to expand the age of patients receiving BMTs while reducing costs^12,13^; and (4) the increasing evidence that haploidentical BMT using posttransplant high-dose Cy has results comparable to those of unrelated-donor BMT without major complications also in LMIC^11,14^ will give a low-cost transplant opportunity to many patients. Cuba, in particular, will certainly benefit from expanding a haploidentical BMT program because transplants from unrelated donors are very rare in Havana due to the disconnection from international marrow-donor registries.

The recent collaboration between UIC and HAH has been focused on building capacity by supporting Cuban investigators in establishing new protocols, such as haplotransplant or autologous transplant in patients older than 65 years, and collecting data and developing a research project that will allow them to analyze and learn from their results.

The retrospective study presented here, despite being limited by relatively few patients and lack of detailed information on GVHD or infectious complications, also represents a rare opportunity to study the outcome of BMT in a country where the high cost of the procedure is not a limiting factor for patients and families. Unlike in many other LMIC,^13,15^ the Cuban government provides full coverage of health care services to Cuban citizens. In fact, Cuban patients with acute leukemia and with an HLA-matched sibling have been offered a standard allo- BMT for over 30 years, whereas for patients who did not have a compatible related donor, an auto-BMT was performed as consolidation chemotherapy, per HAH policy. This approach has allowed us to analyze retrospectively different comparable groups of patients selected only on the basis of donor availability. The similar survival rates we report in auto- and allo-BMT could likely be due to a relatively short follow-up, but these rates could also point to the fact that patients with AML were not selected by disease risk. Physicians in Cuba are now gradually introducing a treatment algorithm in acute leukemias (BMT *v* chemotherapy alone) according to their cytogenetic and molecular profile as these techniques become more accessible. However, this still constitutes a formidable technological challenge yet to be implemented entirely. Challenges of BMT in the setting of LMIC were recently discussed in Nepal within representatives of the UIC GlobalBMT network,^16^ and areas of improvement across countries and specific disease protocols were identified (Table 3). The results achieved through the collaboration of investigators from UIC and HAH have also helped us identify important areas for future research and implementation projects, such as studies on socioeconomic determinants in BMT outcome in Cuba, development of modern diagnostic facilities, training in stem cell cryopreservation, and training in clinical research methods for prospective transplant trials and low-cost cancer treatment studies based on drugs available in Cuba (Table 3).

**Table 3:**
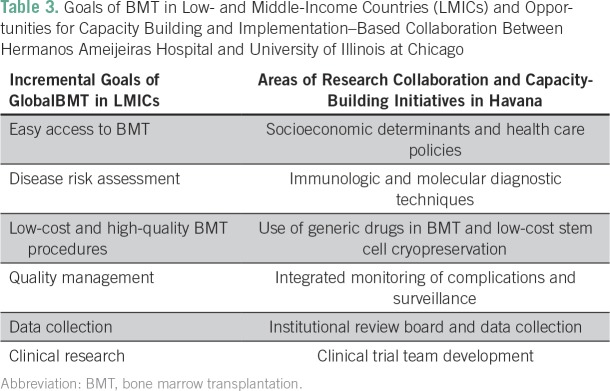
Goals of BMT in Low- and Middle-Income Countries (LMICs) and Opportunities for Capacity Building and Implementation–Based Collaboration Between Hermanos Ameijeiras Hospital and University of Illinois at Chicago

We conclude that centers in high-income countries, such as UIC, can have substantial impact in addressing disparities in LMIC in BMT access^1^ when focused on building capacity and implementation of high-quality and low-cost clinical programs and research infrastructure (Table 3).
